# Two *Cladosporium* Fungi with Opposite Functions to the Chinese White Wax Scale Insect Have Different Genome Characters

**DOI:** 10.3390/jof8030286

**Published:** 2022-03-11

**Authors:** Wei Liu, Shu-Hui Yu, Hong-Ping Zhang, Zuo-Yi Fu, Jia-Qi An, Jin-Yang Zhang, Pu Yang

**Affiliations:** 1 Institute of Highland Forest Science, Chinese Academy of Forestry, Kunming 650224, China; 1.liuwei.2@163.com (W.L.); fuzuoyi000@yeah.net (Z.-Y.F.); aws0306@163.com (J.-Q.A.); 2 Key Laboratory of Breeding and Utilization of Resource Insects of National Forestry and Grassland Administration, Kunming 650224, China; 3 College of Agriculture and Life Sciences, Kunming University, Kunming 650214, China; shuhui19841015@126.com (S.-H.Y.); z2938456055@yeah.net (H.-P.Z.); 4 Faculty of Life Science and Technology, Kunming University of Science and Technology, Kunming 650500, China; jyzhang@kust.edu.cn

**Keywords:** Chinese white wax scale insect, *Cladosporium*, genome, endogensis, pathogen, adaptation

## Abstract

Insects encounter infection of microorganisms, and they also harbor endosymbiosis to participate in nutrition providing and act as a defender against pathogens. We previously found the Chinese white wax scale insect, *Ericerus pela*, was infected and killed by *Cladosporium* sp. (pathogen). We also found it harbored *Cladosporium* sp. (endogensis). In this study, we cultured these two *Cladosporium* fungi and sequenced their genome. The results showed *Cladosporium* sp. (endogensis) has a larger genome size and more genes than *Cladosporium* sp. (pathogen). Pan-genome analysis showed *Cladosporium* sp. (endogensis)-specific genes enriched in pathways related to nutrition production, such as amino acid metabolism, carbohydrate metabolism, and energy metabolism. These pathways were absent in that of *Cladosporium* sp. (pathogen). Gene Ontology analysis showed *Cladosporium* sp. (pathogen)-specific genes enriched in the biosynthesis of asperfuranone, emericellamide, and fumagillin. These terms were not found in that of *Cladosporium* sp. (endogensis). Pathogen Host Interactions analysis found *Cladosporium* sp. (endogensis) had more genes related to loss of pathogenicity and reduced virulence than *Cladosporium* sp. (pathogen). Cytotoxicity assay indicated *Cladosporium* sp. (pathogen) had cytotoxicity, while *Cladosporium* sp. (endogensis) had no cytotoxicity. These characters reflect the adaptation of endosymbiosis to host-restricted lifestyle and the invader of the entomopathogen to the host.

## 1. Introduction

Insects live in various environments and take diverse lifestyles. As a result, they are under the threat of microorganism infection in nature. However, they also establish a mutual relationship with some microorganisms. During the adaptation of insects to different environments and to different pathogenic pressures, microorganisms establish different relationships with insects [1,2,3,4,5]. Even the related microorganism species have different effects on insects [6]. In previous studies, we found that some microorganisms contaminated the honeydew secreted by the females of the Chinese white wax scale insect (*Ericerus pela*) subsequently invade the host and then kill them. We found the lethal microorganisms were mainly *Cladosporium* genus fungi, and we analyzed the changes of microorganism diversity after infection. However, we found some of the endosymbiont fungus of *E. pela* was closely related to the lethal fungus [7]. Why do the related fungi species have two absolutely opposite functions to the same host?

*E. pela* is a traditional resource insect of China and has been bred for more than 1000 years in China. The males secrete white wax and form wax layer covering on their bodies. Females are unable to secrete white wax, instead they form a hard chitin cuticle outside the body to protect themselves [8,9,10]. The females secrete honeydew continually during incubation period, and the honeydew finally forms a huge ball, which adheres to the back of the female adults. The incubation period lasts for more than half a year. The female adults are immobile because of the rudimentary legs. The honeydew is vulnerable to be contaminated by microorganisms especially in the hot and humid regions [7,11]. It has been reported that the honeydew of scale insects and aphids is a source of nourishment to some microorganisms [12]. For *E. pela*, the honeydew they secrete is a good culture medium for *Cladosporium* genus fungi [7].

*Cladosporium* has been reported to be a special genus. They have tremendous adaptability to variety of environments and host. The species of this genus inhabited in a broad range of environments, coving from terrestrial to marine environments. Their hosts range from plants to animals and humans [13,14,15]. *Cladosporium* was reported to infect mammals and human and cause illness [16,17]. They were also reported to cause plant blossom blight and leaf spot [18,19], because they produce a wide range of nature compounds with different bioactive compounds. Some *Cladosporium* were used as biocontrol fungi of pests [13,20,21]. Two studies also showed that, *C. cladosporioides* infection of the host *Cirsium arvense* increased *Cassida rubiginosa* feeding plant leaves [22]. However, *Cladosporium* sp. infection of cauliflower lead to plant resistance to *Spodoptera litura* [23]. There has been no report about the symbiotic relationship between the *Cladosporium* genus and insect host previously.

It has been well recognized that endosymbionts form mutual relationships with host in many insects, especially in the plant phloem-sucking insects (mainly hemipterous insects). They provide essential amino acids and vitamins, which are deficient in the phloem sap, and act as nutrition partners with the insect host. Except for providing nutrition, endosymbiont bacteria also play defensive roles for the host [24,25,26,27]. In aphids, some bacteria are able to produce some antibiotics or toxins, and these compounds protect the hosts from microbial pathogens, parasites, and predators [24,28]. Scale insects are important parts of plant-sucking insects, the endosymbiont fungi are thought to have similar functions with symbiont bacteria in aphids.

There has been convincing evidence indicated the endosymbionts are evolved repeatedly from free-living lineages. The weak pathogen of some symbionts and special location distribution in host imply their environmental origination [24,25]. It was postulated that the endosymbiosis of scale insects is a legacy of the association with microorganisms. The ancestors of scale insect were considered to live under the leaf litters of plants or in the soil. They feed on the root of plants, even the degenerated plants, fungi, and bacteria. The lifestyle leads to the association of a wide range of microorganisms. Maybe they established a certain endosymbiont system before their habitat on the aerial plant parts [29]. However, how the environmental fungi are captured by scale insects and what functions are lost during the formation of a mutual relationship with the host is not clear.

The symbionts lost essential biological functions accompanied by the formation of association. Their genome remained with evolutionary trajectories related to the trend of function loss and transition. The genomes of free-living microorganisms, obligate mutualisms, and facultative mutualisms have different genome sizes and characters. Generally, the obligate microorganisms have a drastically reduced genome. While facultative mutualisms have moderately reduced genomes and have some pathogen features and free-living abilities. The facultative symbionts are thought to be the transition stage from free-living pathogens to mutualisms [24,26,28]. Some of the endogenous microorganisms of *E. pela* could be cultured in vitro and have relatives living freely in the environment. Comparing endogenous microorganisms that have free-living ability with their relatives in nature will provide the opportunities to understand the genetic information related to functional changes that lead to adaptation to insect lifestyle in vivo.

To understand the genomic basis that determines the function adaptation of the two *Cladosporium* species, we cultured the fungi from the honeydew produced by *E. pela* female adults in this study, and at the same time we also cultured the endogenous fungi of *E. pela*. The comparative analyses of genomic character of *Cladosporium* isolated from honeydew and endogenous *Cladosporium* will provide key information to address the question of why the two *Cladosporium* species have drastically different functions to *E. pela* through the view of their adaptation to the host-restricted lifestyle during association evolution between the microorganism and host.

## 2. Materials and Methods

### 2.1. Fungi Cultivation

The honeydew of the infected *E. pela* by *Cladosporium* sp. (pathogen) was collected and washed quickly with 75% ethanol in a 1.5 mL centrifuge tube. Then, the honeydew was dissolved in different concentrations with sterilized water. First, 50 μL of each concentration suspension was spread on the PDA medium, and cultured at 28 °C for about three days. The single colony was streak cultured on a new plate, and after several days the single colony was transferred on new plate. These steps were repeated several times until pure fungus clone was obtained. The cultured fungus was picked up for genome sequencing.

To isolate the endosymbionts of *E. pela*, the eggs were used to isolate endosymbionts. The eggs produced by healthy individuals were washed with 75% ethanol for surface disinfection, and then washed with sterile water. The eggs were homogenized after adding about 100 μL PBS in a 1.5 mL centrifuge tube with a pestle. After short centrifugation, the supernatant was transferred into a new centrifuge tube and dissolved in different concentration. The fungi were cultured in the same way as the honeydew.

### 2.2. DNA Isolation

The colony from one plate was collected by using a cell lifter. The fungus was grided after adding liquid nitrogen. It was incubated at 65 °C for 1 h after adding a CTAB lysis buffer. The supernatant was transferred to a new tube after centrifugation at 10,000 rpm for 5 min. The same volume of mixture of tris-saturated phenol/chloroform/isopentanol (25:24:1) was added to the supernatant, overturned, and blended sufficiently. The mixture was centrifugated at 10,000 rpm for 10 min, and the supernatant was transferred to a new tube and two-thirds of the volume of isopropanol was added. The DNA was precipitated after incubatation at −20 °C for more than 2 h and centrifuged at 12,000 rpm for 15 min. DNA was washed with 75% alcohol.

The internal transcribed spacer (ITS) gene fragment sequence was amplified by using the ITS4 and ITS5 primer pair. Single positive recombinant colonies were sequenced. A nucleotide sequences blastn search showed that the ITS sequence from honeydew fungus DNA has 99.63% similarity with *C. sphaerospermum*, and the ITS sequence from egg fungus DNA has 99.83% similarity with *C. cladosporioides*. The fungus isolated from honeydew was named as *Cladosporium* sp. (pathogen), and the fungus isolated from egg was named as *Cladosporium* sp. (endogensis).

### 2.3. Library Construction and Sequence Filter

The fungus genomic DNA was disrupted by using the ultrasonic DNA disrupter (Covaris, Woburn, MA, USA) to obtain short DNA fragments for library construction. For the 20 kb PacBio sequence library construction, the DNA was disrupted to fragments about 17 kb. After ExoVII digestion, damage repair, end repair, adapter conjunction, enzyme digestion, and fragment selection, the dumbbell-shaped fragment library was obtained. The library quality was validated by Qubit and Agilent 2100. The library was sequenced by PacBio Sequel.

For DNBSEQ library construction, about 1 μg of genomic DNA was disrupted to the fragments of 200–400 bp. The end of the cDNA was repaired, and the A base was added to the end and then the cDNA was connected with the adapter. The conjunction product was amplified by PCR then purified by magnetic bead. The cDNA was melted and became single strings. The single string was cyclized into a circle, and the linear DNA was digested. The circle production was sequenced by DNBSEQ.

### 2.4. Genome Assembly

The subreads were extracted from polymerase reads. The adapters were removed and the subreads with length shorter than 1000 bp were removed.

The subreads were corrected by using the FalconConsensus software [30] or mixed to be corrected by the software Proovread [31]. Highly reliable corrected reads were obtained. The Canu software [32] was used for sequence assembly based on the corrected-reads. The short sequences from BGI sequencing were used for single nucleotide correction of the genome. The reliable assemble sequences were obtained. The SSPACE_Basic_v2.0 software [33] was used to assemble scaffold base on the assemble sequence. The holes in the scaffolds were mended by the pbjelly2 software [34].

### 2.5. Gene Prediction

The assembled genome sequences were aligned with transposon sequence database to identify the repeat sequences. The RepeatMasker software and RepeatProteinMasker software and the corresponding databases were used. For the Denovo prediction, a database was constructed based on its sequences by using the buildXDFDatabase software, then the transposon model was constructed by using the RepeatModeler software according to the database. Transposons were found using the Repeatmasker software according to the model (the software mentioned above were a softwarebag [35]). Finally, the Tandem Repeat Finder software [36] was used to predict tandem repeat sequences.

Non-code RNA was predicted by aligned with rRNA database or predicted by using the RNAmmer software [37]. tRNA regions and tRNA second construction were predicted by using the tRNAscan software [38]. sRNA was predicted by aligning with Rfam database [39] by using the Infernal software [40].

Gene predictions were performed based on the genewise prediction [41], Augustus prediction [42], and GeneMark-ES prediction [43].

### 2.6. Gene Functional Annotation

Gene annotation was performed by amino acid sequences aligned with the database, which included Gene Ontology (GO), Kyoto Encyclopedia of Genes and Genomes (KEGG), Cluster of Orthologous Groups of proteins (COG), Swiss-Prot, Trembl, Non-Redundant Protein Sequence Database (NR), and EggNOG.

The proteins were also aligned with the database of P450, Carbohydrate-Active enZYmes Database (CAZY), Eukaryotic Protein Kinases and Protein Phosphatases (EKPD), the Comprehensive Antibiotic Research Database (CARD), dbCAN (a web server and database for automated carbohydrate-active enzyme annotation), Transporter Classification Database (TCDB), Pathogen–Host Interactions (PHI), and PHOSPHATASE to understand their possible function related to fungal infection.

### 2.7. Core Gene and Specific Gene Analyses

Five sequenced fungi from *Cladosporium* genus (Table 1) and *Cladosporium* sp. (pathogen) and *Cladosporium* sp. (endogensis) were selected for Pan gene analyses. All the predicted proteins from the seven fungal genomes were selected for CD-HIT (v4.6.6) cluster analyses [44,45]. The final gene sets were used as Pan gene sets. The clusters that contains proteins from all the seven fungi were thought to be core gene sets. The proteins that cannot cluster with other sequences were thought to be specific gene sets. The Pan gene sets that exclude core and specific gene sets were thought to be dispensable gene sets.

### 2.8. Phylogenetic Tree Construction

Proteins of the seven fungi were searched by BLAST, and then we removed the redundant proteins using solar software (Version 0.9.6). The aligned gene families were TreeFam clustered using Hcluster_sg software (https://github.com/douglasgscofield/hcluster. accessed on 3 August 2020). The Muscle software [46,47] was used to perform mutule protein sequence alignment of the clustered gene family. The protein alignment results were transferred to coding sequence amino acid mutule sequence alignment results. The TreeBeST software [48] was used to construct phylogenetic tree based on the Muscle [46,47] mutule sequence alignment results. A gene family-based tree was constructed by the NJ method, and a Core-pan gene-based tree was constructed by the PHYML method.

ITS, tef-1α, and act sequences [49] from 68 species (including four outgroups) were aligned separately using MUSCLE and manually adjusted for alignment results. The phylogenetic tree was construct using MEGA (V10.1.7) under the Maximum Likelihood method. The General Time Reversible (GTR) model was used in this tree (Bootstrap = 1000). The rates among sites were set to Gamma Distributed with Invariant Sites (G+I). Other parameters were kept as default values.

### 2.9. Synteny Analyses

The aimed genome sequence of the two fungi were ordered according to the reference fungal genome based on the alignment result of the MUMmer software [50]. The sequences were scaled down according to the three genome sequences length and the X- and *Y*-axis in the two dimensions of the synteny figure were constructed, and the up and down axis in the linear of the synteny figure were also constructed. The aimed fungal protein sets P1 were aligned with reference fungal protein sequence sets P2 by using BLASTp alignment, and the alternate alignment was also performed. The best alignment pair of each protein in the database was selected as the best hit. The protein pairs with consistent result in the two alignments were reserved. The consistent value of the protein pair was the average of the two alignments. The protein pairs were marked in the figure according to their location.

### 2.10. Secondary Metabolite Biosynthetic Gene Clusters Prediction

Secondary metabolite biosynthetic gene clusters in the *Cladosporium* sp. (pathogen) and *Cladosporium* sp. (endogensis) genomes were predicted using the antiSMASH fungal version [51] online (https://fungismash.secondarymetabolites.org/ accessed on 12 Agust 2021).

### 2.11. Cytotoxicity Assay of the Two Cladosporium Species

The fungal colony on the medium dishes was transferred into the liquid medium and shake cultured at 28 °C until the colony filled the liquid medium. Then, several fungal colonies were transferred to new liquid medium and cultured for 7 days. Fungus colonies were absorbed water with filter paper. The *Cladosporium* sp. (pathogen) and *Cladosporium* sp. (endogensis) colonies with the same weight were mashed by liquid nitrogen. After sonic disruption, shaking (1% dimethyl sulfoxide, DMSO), and centrifugation, the supernatant was used for cytotoxicity assay.

*Aedes albopictus* C6/36 cells at the logarithmic growth stage were inoculated in a 96-well plate. The cells were precultured in wells for 24 h, and then 10 µL of solution was added into each well. Each 10 µL of solution contained a gradient volume of dissolved materials of fungi or DMSO as follows: 0 µL, 0.1 µL, 1 µL, 2 µL, 4 µL, 6µL, 8 µL, and 10 µL. Five replicates were set for each solution. After incubation at 28 °C for 24 h, the liquid medium in each well was discarded and 100 µL of fresh medium and then 10 µL of CCK8 (Proteintech Group, Rosemont, IL, USA) were added to each well. After incubation for 1–2 h, the OD value was detected at 450 nm by the microplate reader (Thermo Fisher 3001, Waltham, MA, USA). The cell viability of Aedes albopictus C6/36 cells was calculated according to the formula of the manual (Proteintech Group, Rosemont, IL, USA). Three biological replications were performed. The cell viability results were analyzed using the DPS software. The least significant difference (LSD) test was used and analyses were carried out at the *p* = 0.01 level.

## 3. Results

### 3.1. Genome Assemble Results

**Figure 1:**
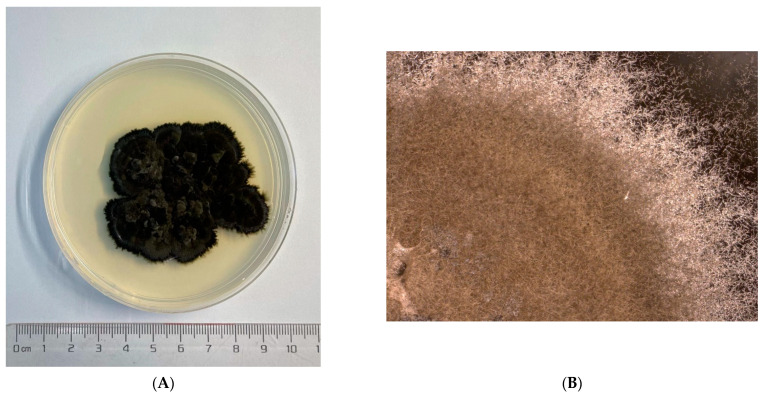

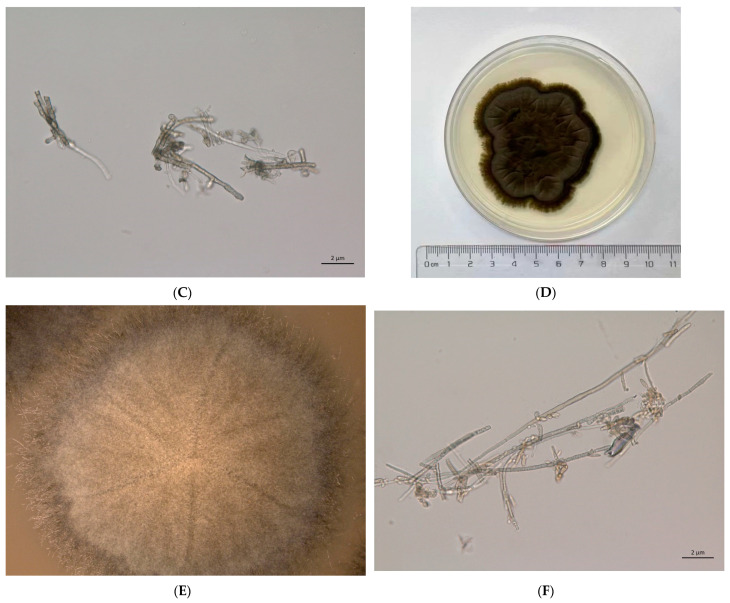
The cultured fungi on the medium dishes at different magnifications. (**A**–**C**): *Cladosporium* sp. (pathogen); (**D**–**F**): *Cladosporium* sp. (endogensis).

**Figure 2:**
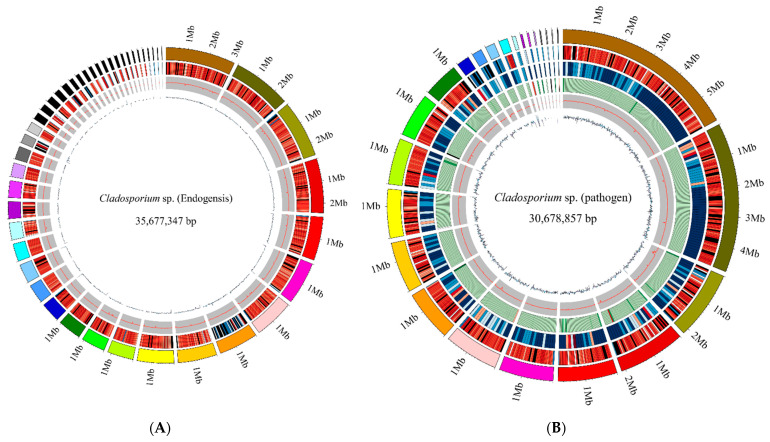
Circular representation of the genomes of *Cladosporium* sp. (pathogen) and *Cladosporium* sp. (endogensis). (**A**) *Cladosporium* sp. (endogensis) genome; (**B**) *Cladosporium* sp. (pathogen) genome.

The cultured *Cladosporium* sp. (pathogen) and *Cladosporium* sp. (endogensis) are shown in Figure 1. A total of 484,886 subreads (4,713,289,584 bp) were obtained from *Cladosporium* sp. (pathogen) with mean length of 9720 bp. The N50 length of the subreads was 11,056 bp. For *Cladosporium* sp. (endogensis), 364,835 subreads number with 3,558,887,476 bp were obtained (Figure 2A). The mean length and N50 of the subreads were 9754 bp and 11,245 bp, respectively (Figure S1, Table 2, Tables S1 and S2).

K-mer = 15 was used to estimate the genome size of the fungi. The genome size of *Cladosporium* sp. (pathogen) was estimated as 35.16 M. The genome size of *Cladosporium* sp. (endogensis) was estimated as 39.18 Mb (Figure S2, Table S3).

The sequences of the *Cladosporium* sp. (pathogen) sample were assembled into 49 contig with total length 30,589 kb. The N50 length of contigs was 1610 kb. The contigs were assembled into 25 scaffolds with total length of 30,679 kb (Figure 2B). The N50 of the scaffold was 1877 kb. The GC content of the *Cladosporium* sp. (pathogen) genome was 53.05% (Figure S1, Table S2).

For *Cladosporium* sp. (endogensis) sample, the sequences were assembled into 55 contig of 35,654 kb with a N50 length 1750 kb. A total of 46 scaffolds were obtained with a total length 35,677 kb. The N50 of the scaffold was 1764 kb. The GC content was 52.58%.

### 3.2. Genome Component Analyses

A total of 1126 kb repeat sequences, which composed 3.67% of the *Cladosporium* sp. (pathogen) genome sequence, were predicted. The repeat sequences included DNA transposon, tandem repeat (TR), long terminal repeat (LTR), non-LTR, and so on. There were 1003 kb transposons, which included DNA, line, LTR, Sine, and others. The transposon composed 3.27% of the genomic sequences (Table 3 and Table S4).

In the *Cladosporium* sp. (endogensis) genome, a total of 4.41% (1572 kb) sequences were predicted as repeat sequences. The transposon was 4.02% of genomic sequences (Table 3 and Table S4).

tRNA, rRNA, sRNA, snRNA, and miRNA were also identified. The RNA composed 0.4% of the *Cladosporium* sp. (pathogen) genomic sequences. For the *Cladosporium* sp. (endogensis) genomic sequence, the RNA was about 0.2% of the genome sequences (Table 4).

A total of 10,930 genes with average length 1613 bp were identified in the *Cladosporium* sp. (pathogen) genome. There were 25,247 exons and 14,317 introns identified in the coding sequences. The average length of exons was 642 bp, and the average length of introns was 99 bp (Table 5).

For the *Cladosporium* sp. (endogensis) genome, a total of 13,522 genes were predicted. The average length was 1570 bp. There were 31,128 exons and 17,606 introns predicted with an average length 631 bp and 90 bp, respectively (Table 5).

The genomic size difference between *Cladosporium* sp. (pathogen) and *Cladosporium* sp. (endogensis) mainly results from the coding sequences of *Cladosporium* sp. (endogensis), which are longer than those of *Cladosporium* sp. (pathogen).

### 3.3. Gene Annotation and Functional Analyses

In the *Cladosporium* sp. (pathogen) genome, 84.67% of the genes were annotated by Nr, 29.15% of the genes were functional annotated by Swissprot, 56.58% of the genes were annotated by GO, and 38.44% of the genes were annotated by KEGG. The genes were also annotated by P450, CAZy, Kinase, CARD, TCDB, and PHI databases. A total of 88.76% of the genes were annotated by the databases (Table 6).

For *Cladosporium* sp. (endogensis) genes, 78.59% of the genes were annotated by Nr, 25.55% of the genes were annotated by Swissprot, 54.21% of the genes were annotated by GO, and 33.78% of the genes were annotated by KEGG. The genes were also annotated by the other databases. In total, 85.53% of the genes were annotated (Table 6).

*Cladosporium* sp. (pathogen) and *Cladosporium* sp. (endogensis) had genes involved in the amino acid biosynthesis pathways (Table S5), while there were some gene differences in the pathways related to “alanine, aspartate, and glutamate metabolism”, “cysteine and methionine metabolism”, “arginine and proline metabolism”, and “histidine metabolism”. They also had gene differences in the pathways of vitamin biosynthesis (Table S6).

### 3.4. Pathogenic Analyses

Eight databases were used for pathogenic analyses. According to the PHI analysis results, *Cladosporium* sp. (endogensis) had more genes related to “loss of pathogenicity”, “reduced virulence”, “reduced virulence increased virulence (hypervirulence) unaffected pathogenicity”, and “reduced virulence loss of pathogenicity unaffected pathogenicity” than *Cladosporium* sp. (pathogen). The late two terms were absent in *Cladosporium* sp. (pathogen) (Table S7).

According to the annotation result from CAZy and dbCAN, *Cladosporium* sp. (endogensis) had more carbohydrate esterases (CE), glycoside hydrolases (GH), and auxiliary activities than *Cladosporium* sp. (pathogen). There was one gene related to “ribosomally synthesized protein/peptide toxins/agonists that target channels and carriers” in *Cladosporium* sp. (pathogen), which was absent in *Cladosporium* sp. (endogensis) (Table S7).

In addition, *Cladosporium* sp. (endogensis) had more p450 genes than *Cladosporium* sp. (pathogen). In the kinase annotation result, *Cladosporium* sp. (endogensis) had more genes related to tyrosine kinase-like (TKL) and tyrosine kinases (TK). One gene of *Cladosporium* sp. (endogensis) was annotated in the CARD annotation result, and no gene of *Cladosporium* sp. (pathogen) was found in CARD annotation result (Table S7).

### 3.5. Specific Gene Analyses

**Figure 3:**
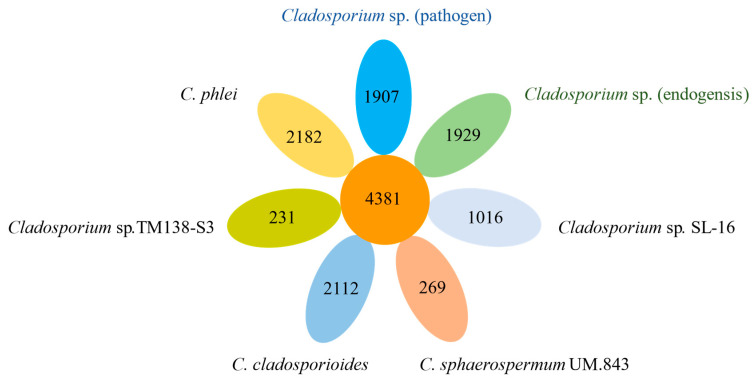
Core and specific genes of *Cladosporium* sp. (pathogen) and *Cladosporium* sp. (endogensis) and other five genomes sequenced of *Cladosporium*.

There were 4381 core genes of the seven fungi identified. For specific genes, *Cladosporium* sp. (pathogen) had 1907 specific genes, and *Cladosporium* sp. (endogensis) had 1929 specific genes (Figure 3 and Figure S3). These species-special genes did not cluster together in a certain scaffold, and it seemed that they were sparsely distributed over the scaffolds.

**Figure 4:**
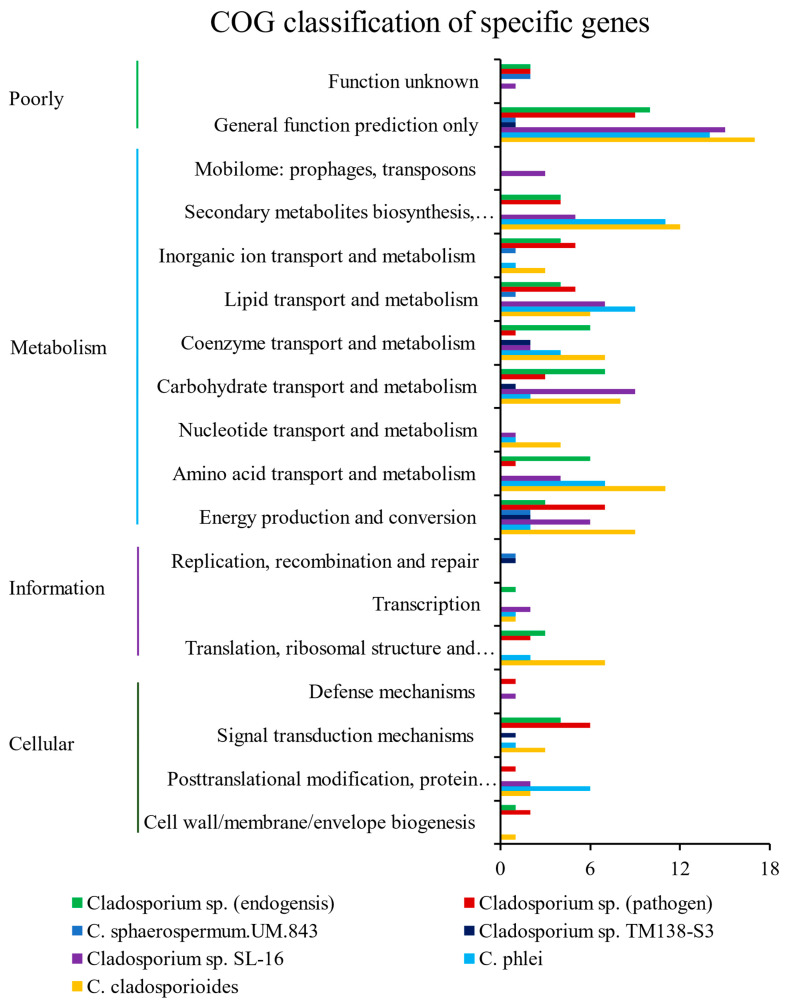
COG analyses of the specific genes of *Cladosporium* sp. (pathogen) and *Cladosporium* sp. (endogensis) and other five *Cladosporium* fungi.

A lot of the specific genes of *Cladosporium* sp. (pathogen) or *Cladosporium* sp. (endogensis) were involved in metabolism (Figure 4). The enriched pathways of specific genes of *Cladosporium* sp. (pathogen) and *Cladosporium* sp. (endogensis) had a great difference in their metabolisms. A certain amount of the specific genes of *Cladosporium* sp. (endogensis) were enriched in the pathways of or related to amino acid metabolism, such as “arginine and proline metabolism”, “histidine metabolism”, “arginine biosynthesis”, “phenylalanine metabolism”, “tyrosine metabolism”, “phenylalanine, tyrosine, and tryptophan biosynthesis”, and “cyanoamino acid metabolism”, while the specific genes of *Cladosporium* sp. (pathogen) did not enrich these amino acid metabolism pathways (Table S8).

Some of the specific genes of *Cladosporium* sp. (endogensis) also enriched in the pathways related to carbohydrate metabolism and energy metabolism, such as “ascorbate and aldarate metabolism”, “pentose phosphate pathway”, “fructose and mannose metabolism”, “nitrogen metabolism”, and “methane metabolism”. These pathways were not found in the *Cladosporium* sp. (pathogen)-specific gene enrichment analyses (Table S8).

However, some of the *Cladosporium* sp. (pathogen)-specific genes were enriched in pathways of “nicotinate and nicotinamide metabolism”, “glycosaminoglycan degradation”, “N-glycan biosynthesis”, “polycyclic aromatic hydrocarbon degradation”, and “dioxin degradation”. Correspondingly, these pathways were not found in the *Cladosporium* sp. (endogensis)-specific gene enrichment analyses (Table S8).

The GO enrichment analysis was also performed, according to the enrichment result, and there was a great difference between the *Cladosporium* sp. (pathogen)-specific genes and *Cladosporium* sp. (endogensis)-specific genes in their biological process. Many *Cladosporium* sp. (pathogen)-specific genes were enriched in the biological process in terms of the biosynthetic process, which involved in “asperfuranone biosynthetic process”, “emericellamide biosynthetic process”, “fumagillin biosynthetic process”, “fumagillin metabolic process”, “monodictyphenone biosynthetic process”, “o-orsellinic acid biosynthetic process”, “sterigmatocystin biosynthetic process”, “tertiary alcohol biosynthetic process”, and “tertiary alcohol metabolic process”, in addition to “transcription, DNA-templated” terms (Figure S4, Table S9). Correspondingly, these terms were not found in the *Cladosporium* sp. (endogensis)-specific gene enrichment analyses. Instead, there were many *Cladosporium* sp. (endogensis)-specific genes enriched in the regulation process.

### 3.6. Secondary Metabolites Analyses

There were many gene clusters responsible for the biosynthesis of betalactone, fungal-RiPP, NRPS, NRPS-like, terpene, and betalactone. These compounds mostly belong to polyketide. The *Cladosporium* sp. (pathogen) genome had 23 gene clusters, and the *Cladosporium* sp. (endogensis) genome had 30 gene clusters. The *Cladosporium* sp. (endogensis) genome had more gene clusters related to betalactone (Table S10).

### 3.7. Phylogenetic Analyses

According to the phylogenetic tree based on the gene family (Figure S5), *Cladosporium* sp. (pathogen) and *Cladosporium* sp. (endogensis) are members of the *Cladosporium* genus. They were located on different branches of the phylogenetic tree (Figure S6).

**Figure 5:**
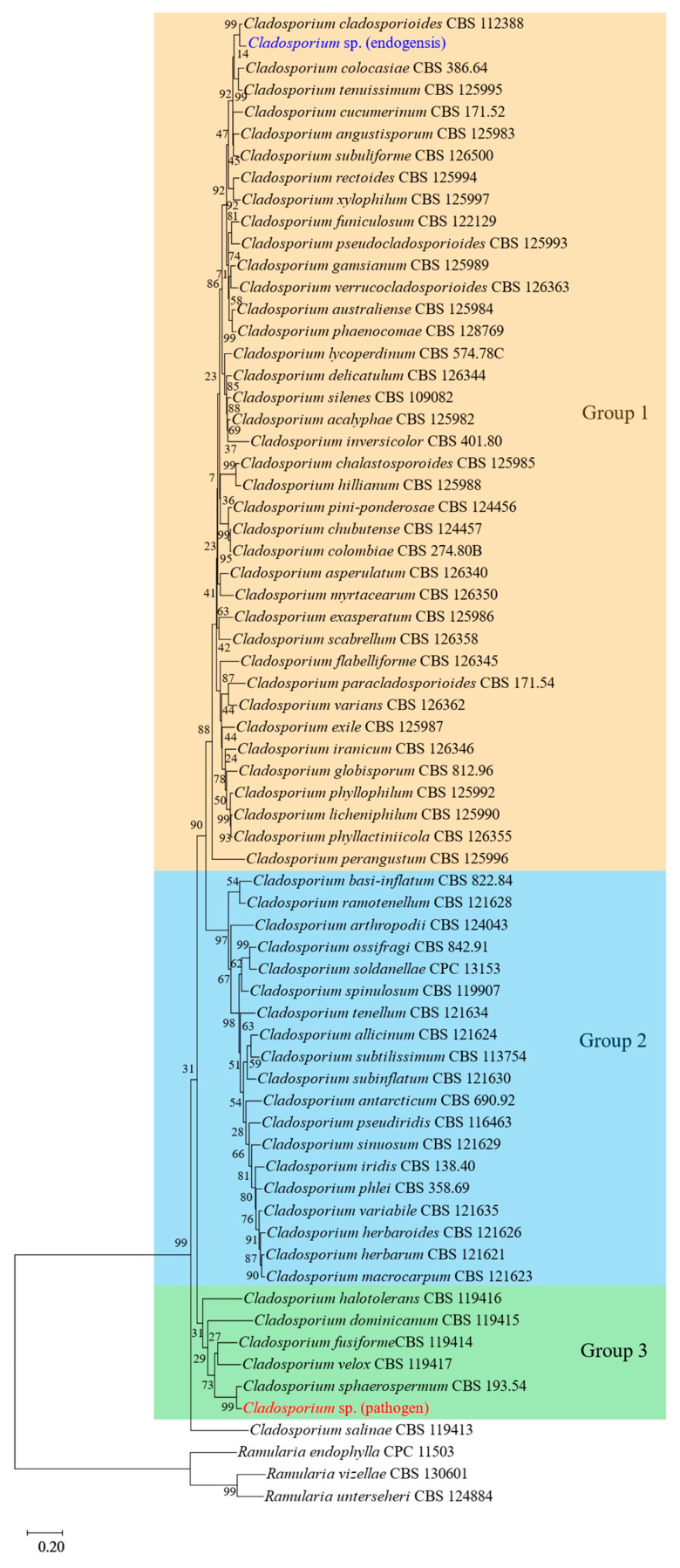
The phylogenetic tree of 68 species constructed by the sequence of ITS, tef-1α, and act. According to Bensch et al. (2012) [49], the *Cladosporium* fungi were divided into three groups: group 1, the *C. cladosporioides* complex; group 2, the *C. herbarum* complex; and group 3, *C. sphaerospermum* complex. The three groups are shown in the figure.

The phylogenetic tree of multiple genes showed that all species of *Cladosporium* were clustered together and divided into three groups. *Cladosporium* sp. (endogensis) formed a sister branch with *C. cladosporioides* and was clustered into the first group. *Cladosporium* sp. (pathogen) formed a sister branch with *C. sphaerospermum* and was clustered into the third group (Figure 5).

### 3.8. Synteny Analyses

Synteny analyses at the amino acid level between *Cladosporium* sp. (pathogen) and *Cladosporium* sp. (endogensis) and the other five species from *Cladosporium* genus revealed the high synteny relationship of *Cladosporium* sp. (pathogen) and *C. phlei*. *Cladosporium* sp. (endogensis) and *C. phlei* also had a high synteny relationship at the amino acid level (Tables S11 and S12, Figures S7–S10).

**Figure 6:**
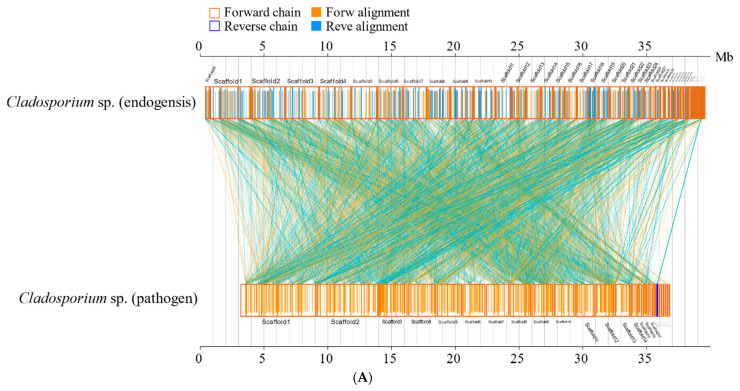

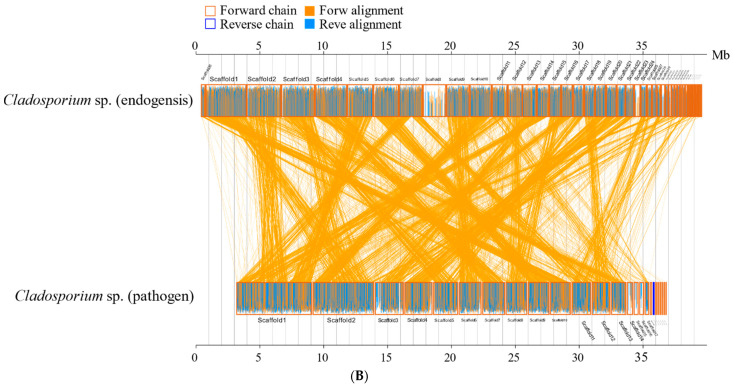
The synteny analyses of *Cladosporium* sp. (endogensis) and *Cladosporium* sp. (pathogen) at the nucleic acid level and the amino acid level. (**A**) nucleic acid level; (**B**): amino acid level.

The synteny analyses’ results also showed that the amino acid sequences of *Cladosporium* sp. (pathogen) and *C. phlei* are highly conserved, and the amino acid sequences of *Cladosporium* sp. (endogensis) and *C. phlei* are highly conserved (Figures S7–S10). The result of the synteny analyses at the nucleic acid level is similar to that at the amino acid level (Figure 6).

### 3.9. Cytotoxicity Analyses

**Figure 7:**
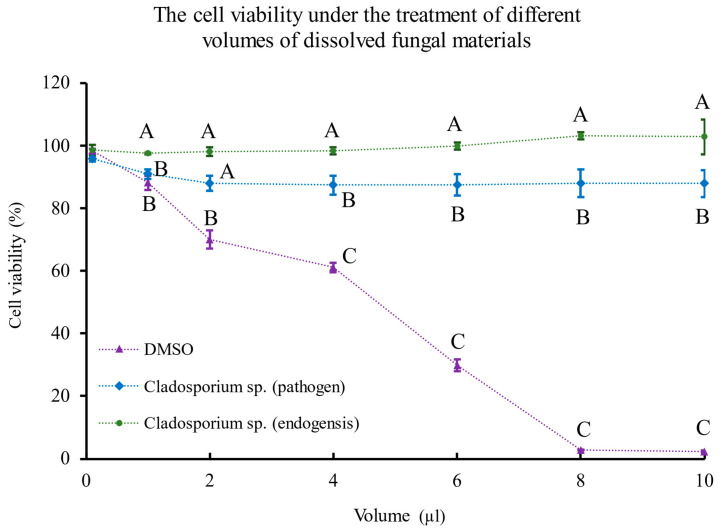
Cytotoxicity assay of *Cladosporium* sp. (pathogen), *Cladosporium* sp. (endogensis), and DMSO using *Aedes albopictus* C6/36 cell line. Three biological replications were performed. Standard deviations (SD) were shown by the error bar at the volume points. Different capital letters of the same volume point represented the significant differences among the three treatments, *p* < 0.01.

According to the cytotoxicity assay results, DMSO inhibited cell viability with the increase of volume, and it almost killed all the cells after the addition of 10 µL. The dissolved materials of *Cladosporium* sp. (pathogen) inhibited the cell viability at a very low volume (1 µL) but did not decrease the cell viability with the volume increase. On the other hand, the dissolved materials of *Cladosporium* sp. (endogensis) could not inhibit cell viability from a low volume to a high volume (Figure 7).

## 4. Discussion

In this study, we sequenced the genome of the two *Cladosporium*. Genome annotation, pan-genome analyses, and secondary metabolite biosynthetic gene cluster prediction were performed. Specific gene analyses found that their species-specific genes were enriched in different pathways. Obviously, *Cladosporium* sp. (endogensis)-specific genes were enriched in the pathways related to amino acid metabolism, carbohydrate metabolism, and energy metabolism. These pathways are important to provide nutrition for the host. These pathways were not found in the enrichment result of *Cladosporium* sp. (pathogen) (Figure S4, Tables S8 and S9). The function differences of *Cladosporium* sp. (pathogen) and *Cladosporium* sp. (endogensis) were thought to result from the differences of their genome characters.

It has been revealed that some bacteria symbionts, such as *Buchnera aphidicola*, *Candidatus Vallotia*, and *Candidatus Profftia*, are able to provide vital vitamins and necessary amino acids for aphids [28,52]. It was shown that bacteria symbionts *Carsonella*_DC and *Candidatus Profftella* in psyllids also provide vitamins and amino acids for the host [26]. The endosymbionts are indispensable for insect hosts, especially for plant-sucking insects, such as aphids, psyllids, whiteflies, and plant hoppers. In this study, we found *Cladosporium* sp. (endogensis) and *Cladosporium* sp. (pathogen) had pathways related to the biosynthesis of amino acids and vitamins (Tables S5 and S6). These genes were important for *Cladosporium* sp. (endogensis) to establish a mutual relationship with *E. pela*.

CAZy and dbCAN annotation showed that *Cladosporium* sp. (endogensis) had more carbohydrate esterases (CE) and glycoside hydrolases (GH) than *Cladosporium* sp. (pathogen) (Table S7). CE and GH were important to deacetylate plant polysaccharides. It is the character of phytopathogenic fungi that they need these enzymes to degenerate polysaccharides and break down plant cell walls to invade plants [53,54]. For sap-sucking insects, they need to pierce the plant phloem of the branch or leaf. In addition, phloem saps have high sugar contents. These food resources tend to result in nutrition deficiency and metabolism burden for insect feeders. The CEs and GHs of *Cladosporium* sp. (endogensis) were thought to play an important role in the phloem sap-sucking lifestyle of *E. pela*.

Compared to *Cladosporium* sp. (pathogen), *Cladosporium* sp. (endogensis) had more genes related to loss or reduction of pathogenicity or virulence (Table S7). “Loss of pathogenicity” and “reduced virulence” represented reduced pathogenicity and virulence against the host [55]. It was inferred that, during evolution, some microorganisms such as the ancestor of *Cladosporium* sp. (endogensis) had the ability to degenerate plant polysaccharides to have endosymbiosis character and act as nutrition partners of *E. pela*. The initial *Cladosporium* in *E. pela* could contaminate the plant phloem sap by the sucking the mouthpart and circulate horizontally in *E. pela*, which could accelerate the spread and colony in the population.

Synteny analyses showed that *Cladosporium* sp. (pathogen) and *Cladosporium* sp. (endogensis) had a significant synteny with *C. phlei* when compared with other *Cladosporium* fungi. *C. phlei* is a phytopathogenic fungus that causes purple eyespot disease, which is a common disease in the timothy plant. In addition, *C. phlei* is intensively studied because it produces the fungal pigment, phleichrome, which is used in photodynamic therapy. This fungal perylenequinone is produced by polyketide synthases (PKS) [56]. Phleichrome has antifungal activity against the fungus *Epichloe typhina* [57]. In this study, secondary metabolite biosynthetic gene cluster analyses showed that *Cladosporium* sp. (pathogen) and *Cladosporium* sp. (endogensis) had gene clusters responsible for the production of some polyketide. However, the cytotoxicity assay showed that *Cladosporium* sp. (endogensis) had no cytotoxicity, while *Cladosporium* sp. (pathogen) showed cytotoxicity even at a low volume. The results were consistent with the endosymbiosis character of *Cladosporium* sp. (endogensis) and the pathogen character of *Cladosporium* sp. (pathogen). In addition, according to the mutual gene phylogenetic analyses, *Cladosporium* sp. (endogensis) is closely related to *C. cladosporioides*. *C. cladosporioides* was identified in the gut of adult beetle *Brachypeplus glaber* [58]. It was shown that *Cladosporium* spp. accounted for 12.0% of the fungal species of the red flour beetle *Tribolium castaneum* [59]. The existence of *Cladosporium* in the insect implied their symbiotic role in the host.

However, it is different for *Cladosporium* sp. (pathogen). It is an entomopathogen of *E. pela* [7]. According to the specific gene analysis, *Cladosporium* sp. (pathogen)-specific genes were enriched in GO terms related to the biosynthesis process of a variety of compounds, such as asperfuranone, emericellamide, fumagillin, o-orsellinic acid, and sterigmatocystin. On the other hand, these terms were absent in the *Cladosporium* sp. (endogensis)-specific gene GO enrichments (Figure S4, Table S9). The genes related to these terms were thought to play an important role in the infection process of *Cladosporium* sp. (pathogen) as they act as a lethal pathogen.

*Cladosporium* fungi distribute in a variety of environments and infect a wide range of hosts [13]. *Cladosporium* fungi are a pathogen for animals and plants. They cause human subcutaneous infection, brain abscess, pulmonary hemorrhage symptoms, and congestion, as well as phaeohyphomycosis. They also cause plant blossom blight and leaf spot [13,16,18]. As an important resource insect, the distribution areas of *E. pela* cover a wide range of climate types from hot and humid climates to cold and dry climates [60,61]. The honeydew produced by females is prone to be contaminated by a variety of pathogens including *Cladosporium* sp. (pathogen) [7]. *E. pela* has been cultured in China for more than 1000 years; however, this period is still very short compare with the evolution process of scale insects. *E. pela* was subjected to the infection of *Cladosporium* sp. (pathogen) during the cultivation and popularization of *E. pela* in some regions. *Cladosporium* sp. (pathogen) is a new pathogen of *E. pela*, and it can be inferred from the reports that the honeydew of scale insects and aphids is a source of nourishment to some microorganisms, especially the Capnodiales order fungi [62]. Thus, *Cladosporium* sp. (pathogen) is a new invader of *E. pela* in some regions during cultivation, which they did not encounter before.

Genome sequence analyses showed that *Cladosporium* sp. (endogensis) fungi have a larger genome size than *Cladosporium* sp. (pathogen), and the genome size is also larger than the other *Cladosporium* species that have been genome sequenced. It was different with genomic analyses of pathogenic bacteria and bacterial symbiosis in aphids. In aphids, the three free-living *Serratia* strains that are pathogenic to aphid hosts have a larger genome size than the facultative or obligate *Serratia* strains. It was thought that the transition of *Serratia* strains from a free-living stage to a host-restricted stage, comparing the reduction of genome size, mainly results from the losses of metabolic pathways or genes related to metabolism [24]. However, in plants, some biotrophic pathogens have increased their genome size. Spanu (2012) holds that the increase or decrease in genome size was thought to be driven especially by the diversification and creation of effector genes [63]. It was thought that *Cladosporium* sp. (endogensis) fungi increased their genome size and formed a new genomic character to facilitate the mutual relationship with the *E. pela* host. While *Cladosporium* sp. (pathogen), acting as a pathogenic fugus, maintained the character of invader and lived free in nature.

## Figures and Tables

**Table 1:** The species name and accession number of five genome sequenced fungi from *Cladosporium* genus used in this study.

Species Name	Accession Number in GenBank
*Cladosporium phlei*	QZFA00000000
*Cladosporium sphaerospermum*.UM.843	AIIA02
*Cladosporium* sp.SL-16	PEGC00000000.1
*Cladosporium* sp.TM138-S3	JAAQHG000000000.1
*Cladosporium cladosporioides*	NOXB01

**Table 2:** Scaffold and contig analyses of *Cladosporium* sp. (pathogen) and *Cladosporium* sp. (endogensis) genome assembly.

Sequence Type	Sample	Total Number	Total Length (bp)	N50 Length (bp)	N90 Length (bp)	Max Length (bp)	Min Length (bp)	Gap Number (bp)	GC Content
Scaffold	*Cladosporium* sp. (pathogen)	25	30,678,857	1,877,121	1,391,070	5,850,328	22,622	89,418	53.05%
	*Cladosporium* sp. (endogensis)	46	35,677,347	1,764,234	4,247,99	3,136,047	11,843	23,760	52.58%
Contig	*Cladosporium* sp. (pathogen)	49	30,589,439	1,610,294	428,466	3,437,199	22,329	-	53.05%
	*Cladosporium* sp. (endogensis)	55	35,653,587	1,749,682	37,981,6	25,907,13	11,843	-	52.58%

**Table 3:** The statistic of repeat sequence from different predication methods.

Method	Sample	Repeat Size (bp)	Percent in Genome
Repbase	*Cladosporium* sp. (pathogen)	240,641	0.7844%
	*Cladosporium* sp. (endogensis)	252,517	0.7078%
ProMask	*Cladosporium* sp. (pathogen)	490,596	1.5991%
	*Cladosporium* sp. (endogensis)	525,112	1.4718%
denovo	*Cladosporium* sp. (pathogen)	758,713	2.4731%
	*Cladosporium* sp. (endogensis)	1,150,874	3.2258%
TRF	*Cladosporium* sp. (pathogen)	155,625	0.5073%
	*Cladosporium* sp. (endogensis)	190746	0.5346%
Total	*Cladosporium* sp. (pathogen)	1,125,929	3.67%
	*Cladosporium* sp. (endogensis)	1,572,493	4.4075%

**Table 4:** None-code RNA in the genome of *Cladosporium* sp. (pathogen) and *Cladosporium* sp. (endogensis).

Type	Sample	Copy	Average Length	Total Length	Percent in Genome
tRNA	*Cladosporium* sp. (pathogen)	221	92	20,406	0.0665%
	*Cladosporium* sp. (endogensis)	274	91	25,030	0.0702%
rRNA (by denovo prediction)	*Cladosporium* sp. (pathogen)	82	1237	101,498	0.3308%
	*Cladosporium* sp. (endogensis)	80	465	37,235	0.1044%
sRNA	*Cladosporium* sp. (pathogen)	10	58	581	0.0019%
	*Cladosporium* sp. (endogensis)	46	88	4044	0.0113%
snRNA	*Cladosporium* sp. (pathogen)	4	58	232	0.0008%
	*Cladosporium* sp. (endogensis)	34	113	3848	0.0108%
miRNA	*Cladosporium* sp. (pathogen)	0	0	0	0
	*Cladosporium* sp. (endogensis)	64	60	3877	0.0109%

**Table 5:** The exons and coding sequence statistic of genes of *Cladosporium* sp. (pathogen) and *Cladosporium* sp. (endogensis).

Type	Sample	Total Number	Total Length (bp)	Average Length (bp)	Percent in Genome
Gene	*Cladosporium* sp. (pathogen)	10,930	17,633,154	1613	57.48%
	*Cladosporium* sp. (endogensis)	13,522	21,235,259	1570	59.52%
Exons	*Cladosporium* sp. (pathogen)	25,247	16,208,934	642	52.83%
	*Cladosporium* sp. (endogensis)	31,128	19,653,616	631	55.09%
CDS	*Cladosporium* sp. (pathogen)	10,930	16,208,934	1483	52.83%
	*Cladosporium* sp. (endogensis)	13,522	19,653,616	1453	55.09%
Introne	*Cladosporium* sp. (pathogen)	14,317	1,424,220	99	4.64%
	*Cladosporium* sp. (endogensis)	17,606	1,581,643	90	4.43%

**Table 6:** The statistic of gene classification and functional annotation of *Cladosporium* sp. (pathogen) and *Cladosporium* sp. (endogensis) using different databases.

	Number		Percent	
Annotation Database	*Cladosporium* sp. (Pathogen)	*Cladosporium* sp. (Endogensis)	*Cladosporium* sp. (Pathogen)	*Cladosporium* sp. (Endogensis)
Total	10,930	13,522		
NR	9255	10,627	84.67%	78.59%
SWISSPROT	3187	3456	29.15%	25.55%
GO	6185	7331	56.58%	54.21%
KEGG	4202	4569	38.44%	33.78%
KOG	2151	2237	19.67%	16.54%
COG	1337	1506	12.23%	11.13%
NOG	7799	8835	71.35%	65.33%
IPR	8405	10,064	76.89%	74.42%
CAZY	433	544	3.96%	4.02%
DBCAN	583	725	5.33%	5.36%
PHI	1143	1202	10.45%	8.89%
P450	1762	2202	16.12%	16.28%
KINASE	115	133	1.05%	0.98%
CARD	0	1	0.00%	0.00%
TCDB	565	614	5.16%	4.54%
PHOSPHATASE	34	36	0.31%	0.26%
Over All	9702	11,566	88.76%	85.53%

## Data Availability

Not applicable.

## Supplementary Materials

The following supporting information can be downloaded at: https://www.mdpi.com/article/10.3390/jof8030286/s1, Figure S1. GC content and sequencing depth of *Cladosporium* sp. (pathogen) and *Cladosporium* sp. (endogensis) genome. A: *Cladosporium* sp. (pathogen); B: *Cladosporium* sp. (endogensis). Figure S2. K-mer analyses of genome size of *Cladosporium* sp. (pathogen) and *Cladosporium* sp. (endogensis). The parameters used were shown in the figures. A: *Cladosporium* sp. (pathogen); B: *Cladosporium* sp. (endogensis). Figure S3. COG classification of core genes and indispensable genes in of the seven *Cladosporium* fungi. Figure S4. Directed acyclic graph of GO-biological process enrichment of *Cladosporium* sp. (pathogen) specific genes. Figure S5. Orthologs in *Cladosporium* sp. (pathogen) and *Cladosporium* sp. (endogensis) and other five genome sequenced *Cladosporium* fungi. Figure S6. The phylogenetic trees based on gene family and core genes of *Cladosporium* sp. (pathogen) and *Cladosporium* sp. (endogensis) and other five *Cladosporium* fungi. Gene family-based tree was constructed by NJ method, and Core-pan gene-based tree was constructed by PHYML method. A: The phylogenetic tree based on gene family of the seven *Cladosporium* fungi; B: The phylogenetic tree based on core genes of the seven *Cladosporium* fungi. Figure S7. The synteny analysis of *Cladosporium* sp. (pathogen) with other five genome sequenced *Cladosporium* fungi at nucleic acid level. Figure S8. The synteny analysis of *Cladosporium* sp. (pathogen) with other five genome sequenced *Cladosporium* fungi at amino acid level. Figure S9. The synteny analysis of *Cladosporium* sp. (endogensis) with other five genome sequenced *Cladosporium* fungi at nucleic acid level. Figure S10. The synteny analysis of *Cladosporium* sp. (endogensis) with other five genome sequenced *Cladosporium* fungi at amino acid level; Table S1. The statistic of number and length of the subreads from *Cladosporium* sp. (pathogen) and *Cladosporium* sp. (endogensis) produced by Pac-bio sequencing. Table S2. Statistic of coverage and depth of scaffolds of *Cladosporium* sp. (pathogen) and *Cladosporium* sp. (endogensis). Table S3. K-mer analysis of the reads of *Cladosporium* sp. (pathogen) and *Cladosporium* sp. (endogensis). 15-mer was used and genome size was estimated. Table S4. The statistic and classification of transposons in the genome of *Cladosporium* sp. (pathogen) and *Cladosporium* sp. (endogensis) predicted by different methods. Table S5. The genes and pathways involved in amino acid biosynthesis in the genome of *Cladosporium* sp. (pathogen) and *Cladosporium* sp. (endogensis). Table S6. The genes and pathways invovled in the biosynthesis and metabolism of vitamins in the genome of *Cladosporium* sp. (pathogen) and *Cladosporium* sp. (endogensis). Table S7. Pathogenic analyses and gene annotation of *Cladosporium* sp. (pathogen) and *Cladosporium* sp. (endogensis) genome from different database. Table S8. KEGG enrichment analyses of specific genes of *Cladosporium* sp. (pathogen) and *Cladosporium* sp. (endogensis). Table S9. Biological process of GO enrichment analysis of specific genes of *Cladosporium* sp. (pathogen) and *Cladosporium* sp. (endogensis). Table S10. Secondary metabolites analyses of *Cladosporium* sp. (pathogen) and *Cladosporium* sp. (endogensis) using the antiSMASH fungal version. Table S11. Statistics of synteny analysis of *Cladosporium* sp. (pathogen) with other *Cladosporium* fungi at nucleic acid level. Table S12. Statistics of synteny analysis of *Cladosporium* sp. (endogensis) with other *Cladosporium* fungi at nucleic acid level.
