# Supplementary material for: Two Cladosporium Fungi with Opposite Functions to the Chinese White Wax Scale Insect Have Different Genome Characters
Source: J Fungi (Basel). 2022 Mar 11;8(3):286. doi: 10.3390/jof8030286 (PMC8949958; doi:10.3390/jof8030286)
Supplement: Supplementary file 1 [file jof-08-00286-s001.zip › jof-1624251-supplementary/Additional table titles and figure legends.pdf]

**Additional tables:**

Table S1. The statistic of number and length of the subreads from *Cladosporium* sp. (pathogen) and *Cladosporium* sp. (endogensis) produced by Pac-bio sequencing.

Table S2. Statistic of coverage and depth of scaffolds of *Cladosporium* sp. (pathogen) and *Cladosporium* sp. (endogensis).

Table S3. K-mer analysis of the reads of *Cladosporium* sp. (pathogen) and *Cladosporium* sp. (endogensis). 15-mer was used and genome size was estimated.

Table S4. The statistic and classification of transposons in the genome of *Cladosporium* sp. (pathogen) and *Cladosporium* sp. (endogensis) predicted by different methods.

Table S5. The genes and pathways involved in amino acid biosynthesis in the genome of *Cladosporium* sp. (pathogen) and *Cladosporium* sp. (endogensis).

Table S6. The genes and pathways involed in the biosynthesis and metabolism of vitamins in the genome of *Cladosporium* sp. (pathogen) and *Cladosporium* sp. (endogensis).

Table S7. Pathogenic analyses and gene annotation of *Cladosporium* sp. (pathogen) and *Cladosporium* sp. (endogensis) genome from different database.

Table S8. KEGG enrichment analyses of specific genes of *Cladosporium* sp. (pathogen) and *Cladosporium* sp. (endogensis).

Table S9. Biological process of GO enrichment analysis of specific genes of *Cladosporium* sp. (pathogen) and *Cladosporium* sp. (endogensis).

Table S10. Secondary metabolites analyses of *Cladosporium* sp. (pathogen) and *Cladosporium* sp. (endogensis) using the antiSMASH fungal version.

Table S11. Statistics of synteny analysis of *Cladosporium* sp. (pathogen) with other *Cladosporium* fungi at nucleic acid level.

Table S12. Statistics of synteny analysis of *Cladosporium* sp. (endogensis) with other *Cladosporium* fungi at nucleic acid level.

**Additional figure legends:**

Figure S1. GC content and sequencing depth of *Cladosporium* sp. (pathogen) and *Cladosporium* sp. (endogensis) genome. A: *Cladosporium* sp. (pathogen); B: *Cladosporium* sp. (endogensis).

Figure S2. K-mer analyses of genome size of *Cladosporium* sp. (pathogen) and *Cladosporium* sp.

(endogensis). The parameters used were shown in the figures. A: *Cladosporium* sp. (pathogen); B: *Cladosporium* sp. (endogensis).

Figure S3. COG classification of core genes and indispensable genes of the seven *Cladosporium* fungi.

Figure S4. Directed acyclic graph of GO-biological process enrichment of *Cladosporium* sp. (pathogen) specific genes.

Figure S5. Orthologs in *Cladosporium* sp. (pathogen) and *Cladosporium* sp. (endogensis) and other five genome sequenced *Cladosporium* fungi.

Figure S6. The phylogenetic trees based on gene family and core genes of *Cladosporium* sp. (pathogen) and *Cladosporium* sp. (endogensis) and other five *Cladosporium* fungi. Gene family-based tree was constructed by NJ method, and Core-pan gene-based tree was constructed by PHYML method. A: The phylogenetic tree based on gene family of the seven *Cladosporium* fungi; B: The phylogenetic tree based on core genes of the seven *Cladosporium* fungi.

Figure S7. The synteny analysis of *Cladosporium* sp. (pathogen) with other five genome sequenced *Cladosporium* fungi at nucleic acid level.

Figure S8. The synteny analysis of *Cladosporium* sp. (pathogen) with other five genome sequenced *Cladosporium* fungi at amino acid level.

Figure S9. The synteny analysis of *Cladosporium* sp. (endogensis) with other five genome sequenced *Cladosporium* fungi at nucleic acid level.

Figure S10. The synteny analysis of *Cladosporium* sp. (endogensis) with other five genome sequenced *Cladosporium* fungi at amino acid level.
