# Supplementary material for: Two Cladosporium Fungi with Opposite Functions to the Chinese White Wax Scale Insect Have Different Genome Characters
Source: J Fungi (Basel). 2022 Mar 11;8(3):286. doi: 10.3390/jof8030286 (PMC8949958; doi:10.3390/jof8030286)
Supplement: Supplementary file 1 [file jof-08-00286-s001.zip › jof-1624251-supplementary/additional tables/Table S1, S3 and S4.docx]

Table S1. The statistic of number and length of the subreads from *Cladosporium* sp. (pathogen) and *Cladosporium* sp. (endogensis) produced by Pac-bio sequencing.

| Sample | Valid ZWM Number | Subreads Number | Total Bases (bp) | Mean Length (bp) | N50 (bp) | N90 (bp) | Max Length (bp) | Min Length (bp) |
| --- | --- | --- | --- | --- | --- | --- | --- | --- |
| *Cladosporium* sp. (pathogen) | 85848 | 484886 | 4713289584 | 9720 | 11056 | 6815 | 179946 | 1000 |
| *Cladosporium* sp. (endogensis) | 67262 | 364835 | 3558887476 | 9754 | 11245 | 6789 | 189243 | 1000 |

Table S3. K-mer analysis of the reads of *Cladosporium* sp. (pathogen) and *Cladosporium* sp. (endogensis). 15-mer was used and genome size was estimated.

| Sample | K-mer | K-mer numbers (Mbp) | Pk depth | Genome size (Mbp) | Genome depth |
| --- | --- | --- | --- | --- | --- |
| *Cladosporium* sp. (pathogen) | 15 | 1057.74 | 30 | 35.16 | 33.18 |
| *Cladosporium* sp. (endogensis) | 15 | 1178.63 | 30 | 39.18 | 33.18 |

Table S4. The statistic and classification of transposons in the genome of *Cladosporium* sp. (pathogen) and *Cladosporium* sp. (endogensis) predicted by different methods.

| Method | Sample | Type | DNA | LINE | LTR | SINE | Other | Unknown | Total |
| --- | --- | --- | --- | --- | --- | --- | --- | --- | --- |
| Repbase TEs | *Cladosporium* sp. (pathogen) | Length (bp) | 61229 | 62677 | 117364 | 3975 | 0 | 1119 | 240641 |
|  |  | Percent in Genome (%) | 0.1996 | 0.2043 | 0.3826 | 0.013 | 0 | 0.0036 | 0.7844 |
|  | *Cladosporium* sp. (endogensis) | Length (bp) | 79730 | 87145 | 84457 | 5355 | 0 | 1599 | 252517 |
|  |  | Percent in Genome (%) | 0.2235 | 0.2443 | 0.2367 | 0.015 | 0 | 0.0045 | 0.7078 |
| ProteinMask TEs | *Cladosporium* sp. (pathogen) | Length (bp) | 197804 | 128688 | 164104 | 0 | 0 | 0 | 490596 |
|  |  | Percent in Genome (%) | 0.6448 | 0.4195 | 0.5349 | 0 | 0 | 0 | 1.5991 |
|  | *Cladosporium* sp. (endogensis) | Length (bp) | 201495 | 242335 | 81459 | 0 | 0 | 0 | 525112 |
|  |  | Percent in Genome (%) | 0.5648 | 0.6792 | 0.2283 | 0 | 0 | 0 | 1.4718 |
| Denovo TEs | *Cladosporium* sp. (pathogen) | Length (bp) | 192227 | 105825 | 176904 | 1515 | 0 | 285552 | 758713 |
|  |  | Percent in Genome (%) | 0.6266 | 0.3449 | 0.5766 | 0.0049 | 0 | 0.9308 | 2.4731 |
|  | *Cladosporium* sp. (endogensis) | Length (bp) | 105214 | 215081 | 184613 | 1663 | 0 | 651922 | 1150874 |
|  |  | Percent in Genome (%) | 0.2949 | 0.6029 | 0.5175 | 0.0047 | 0 | 1.8273 | 3.2258 |
| Combined TEs | *Cladosporium* sp. (pathogen) | Length (bp) | 303017 | 180826 | 274559 | 4724 | 0 | 286463 | 1002684 |
|  |  | Percent in Genome (%) | 0.9877 | 0.5894 | 0.8949 | 0.0154 | 0 | 0.9337 | 3.2683 |
|  | *Cladosporium* sp. (endogensis) | Length (bp) | 260030 | 310983 | 265144 | 6222 | 0 | 653521 | 1433143 |
|  |  | Percent in Genome (%) | 0.7288 | 0.8717 | 0.7432 | 0.0174 | 0 | 1.8318 | 4.017 |
