# Supplementary figures and images for: Two Cladosporium Fungi with Opposite Functions to the Chinese White Wax Scale Insect Have Different Genome Characters
Source: J Fungi (Basel). 2022 Mar 11;8(3):286. doi: 10.3390/jof8030286 (PMC8949958; doi:10.3390/jof8030286)

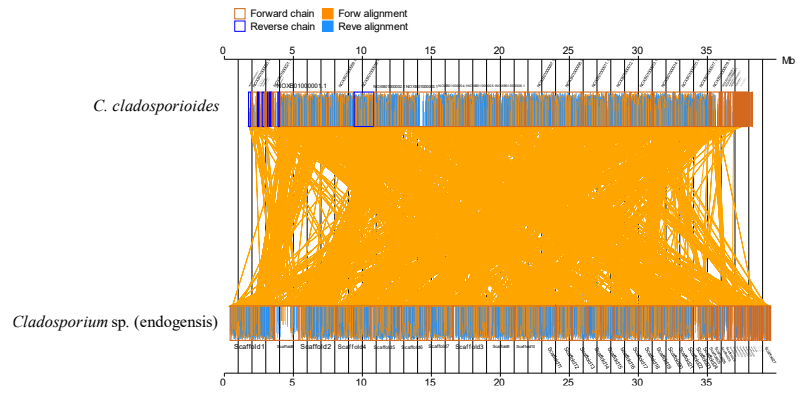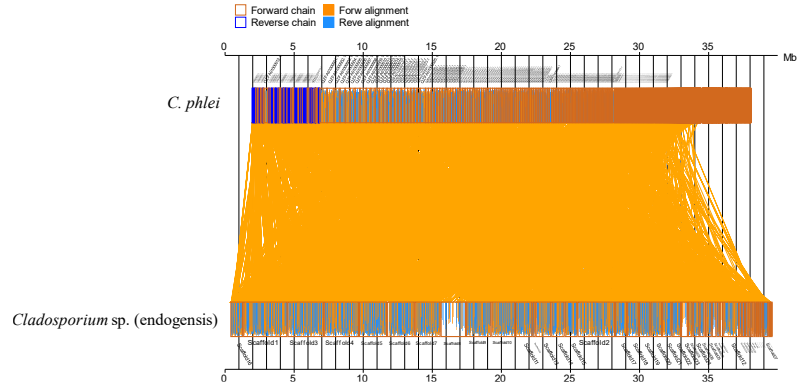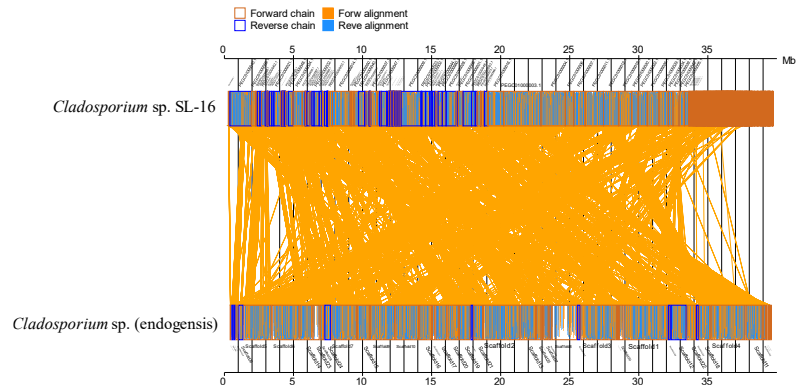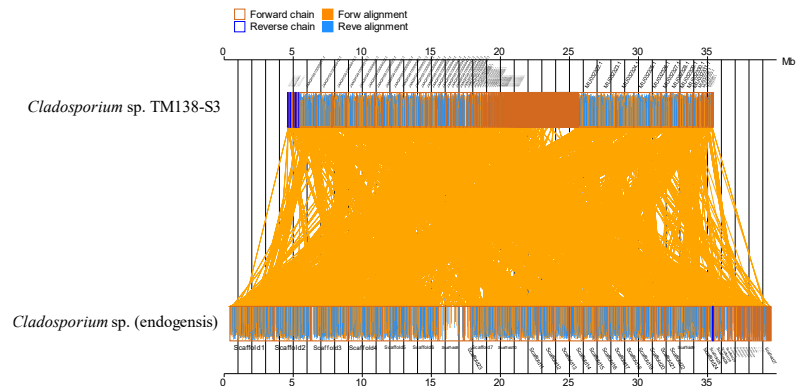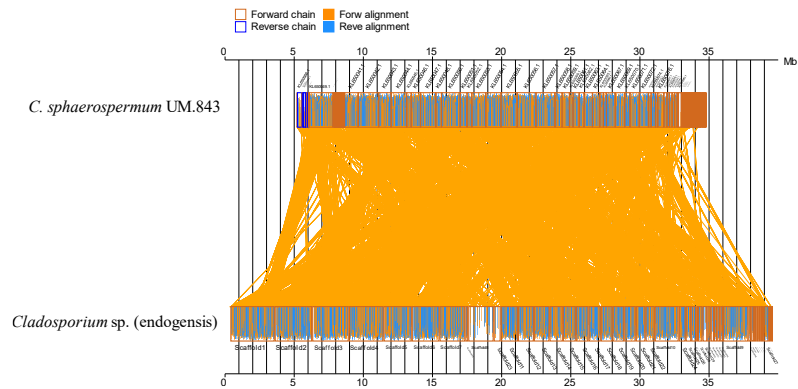

Supplement: Supplementary file 1 [file jof-08-00286-s001.zip › jof-1624251-supplementary/additional figures/Figure S10.pdf]

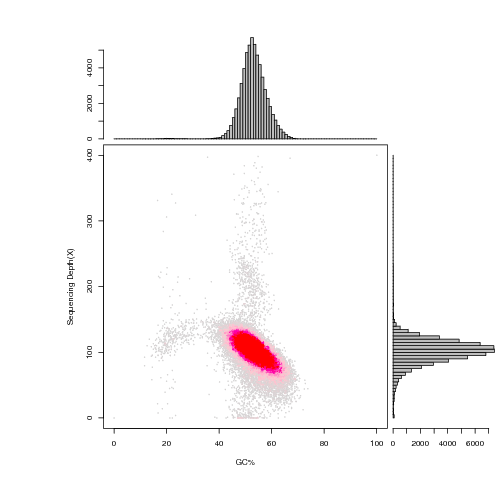

Supplement: Supplementary file 1 [file jof-08-00286-s001.zip › jof-1624251-supplementary/additional figures/Figure S1a.png]

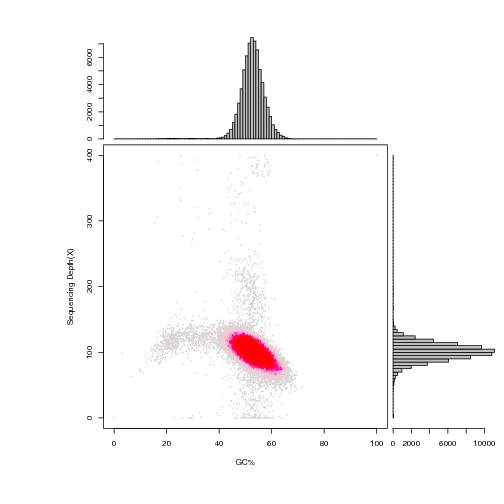

Supplement: Supplementary file 1 [file jof-08-00286-s001.zip › jof-1624251-supplementary/additional figures/Figure S1b.png]

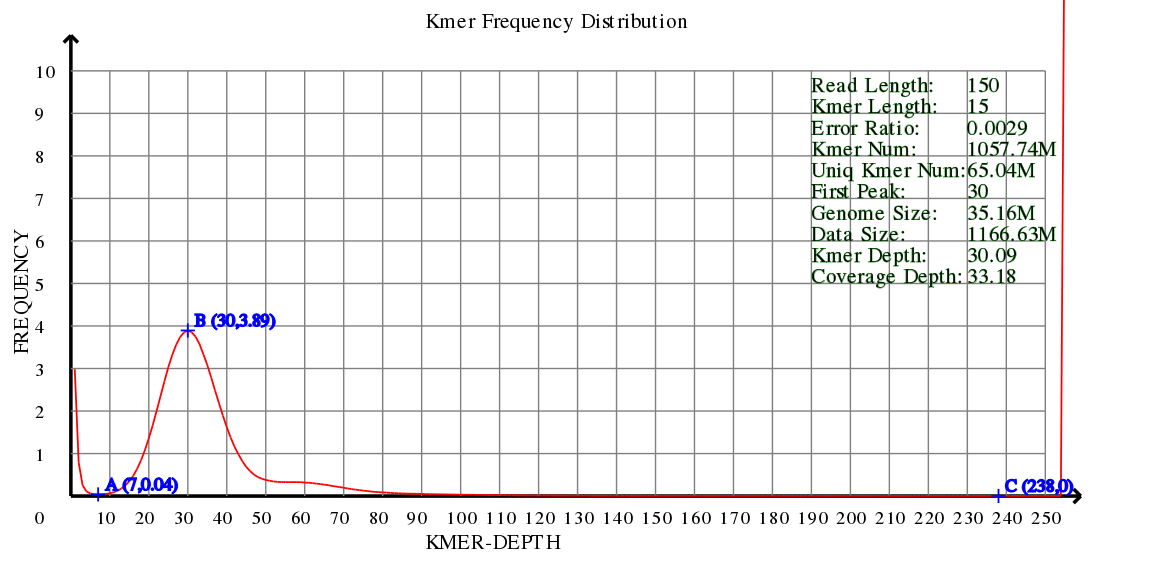

Supplement: Supplementary file 1 [file jof-08-00286-s001.zip › jof-1624251-supplementary/additional figures/Figure S2a.png]

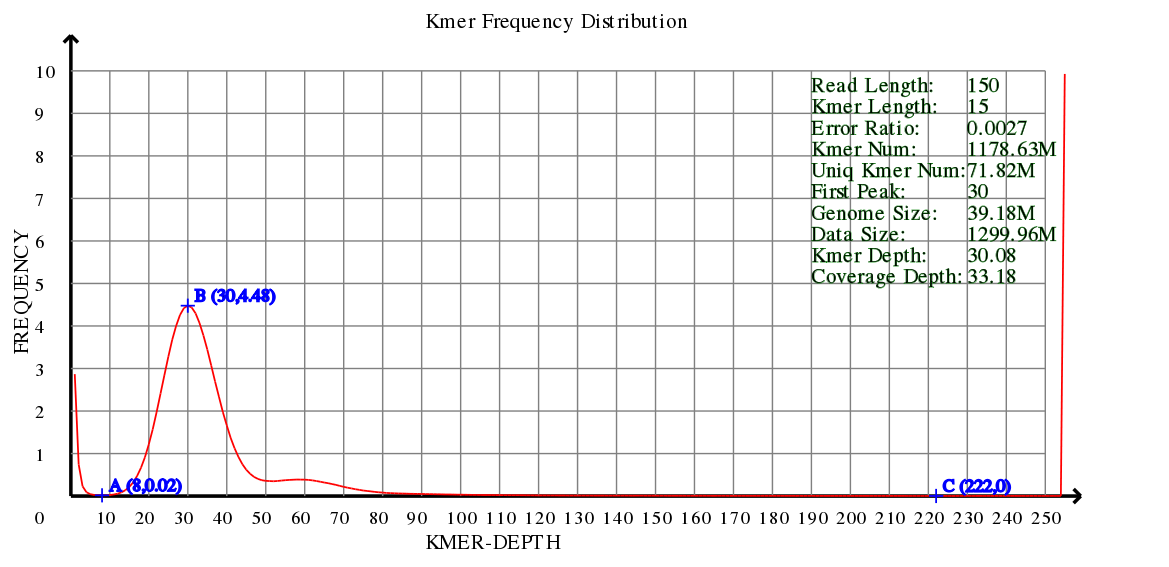

Supplement: Supplementary file 1 [file jof-08-00286-s001.zip › jof-1624251-supplementary/additional figures/Figure S2b.png]

## COG Classification of core and dispensable genes

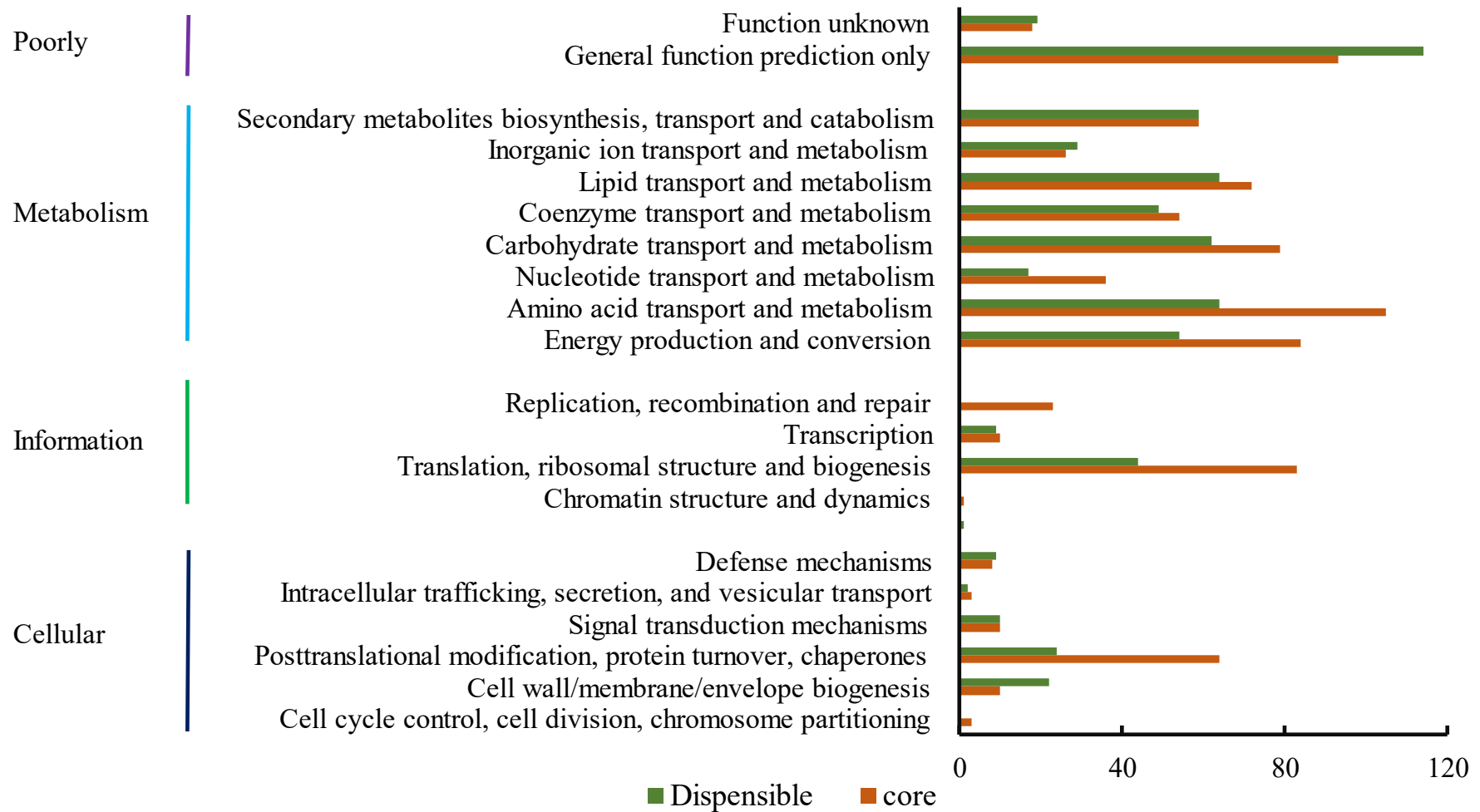

Supplement: Supplementary file 1 [file jof-08-00286-s001.zip › jof-1624251-supplementary/additional figures/Figure S3.pdf]

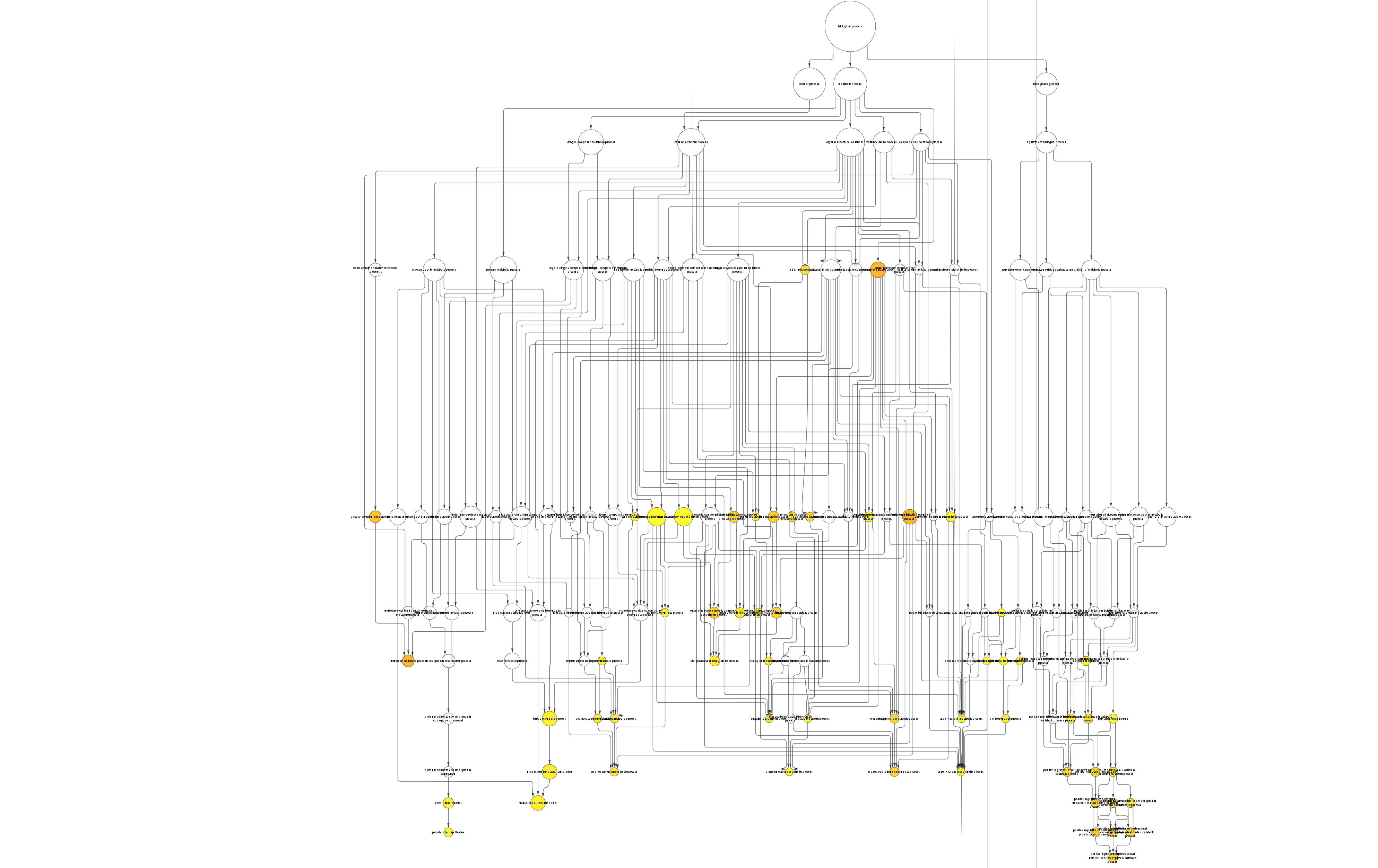

Supplement: Supplementary file 1 [file jof-08-00286-s001.zip › jof-1624251-supplementary/additional figures/Figure S4.png]

The ortholog numbers of different *Cladosporium*

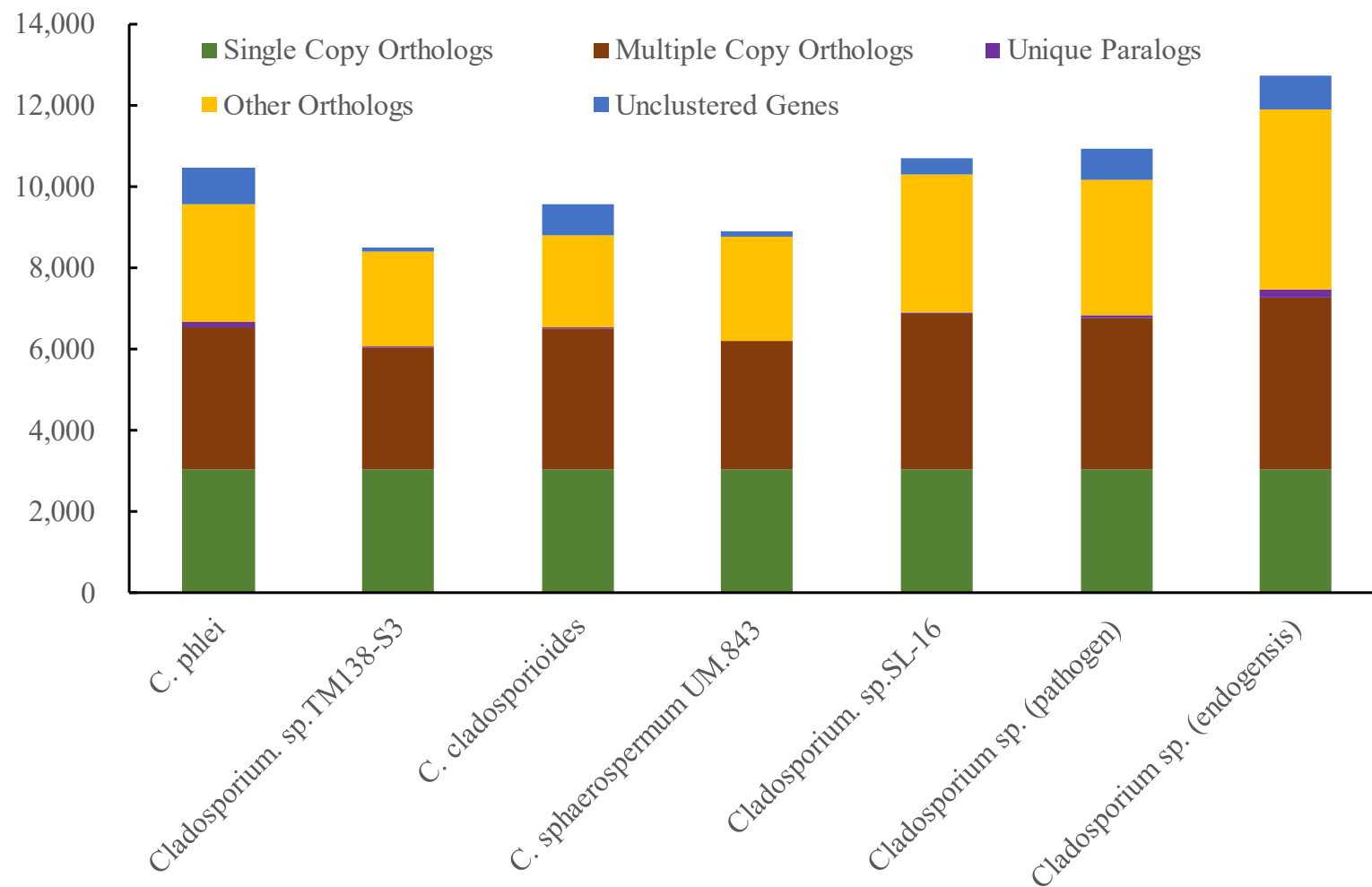

Supplement: Supplementary file 1 [file jof-08-00286-s001.zip › jof-1624251-supplementary/additional figures/Figure S5.pdf]

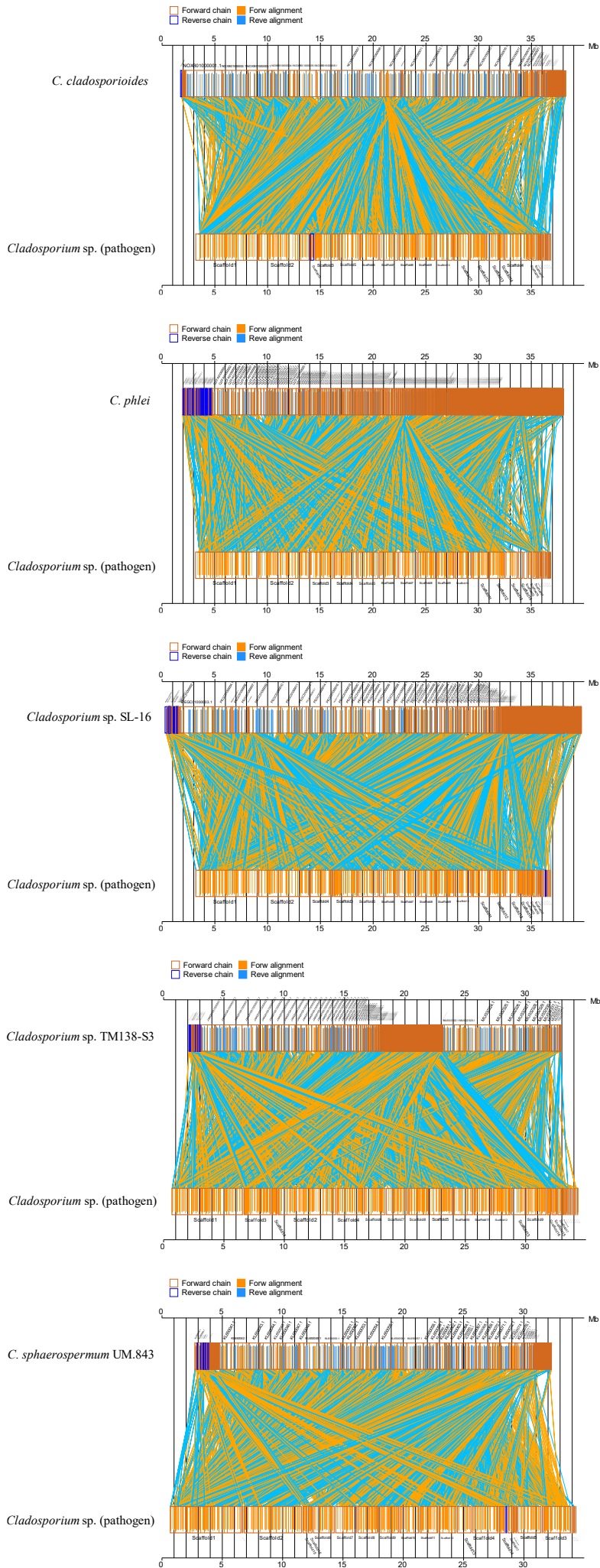

Supplement: Supplementary file 1 [file jof-08-00286-s001.zip › jof-1624251-supplementary/additional figures/Figure S7.pdf]

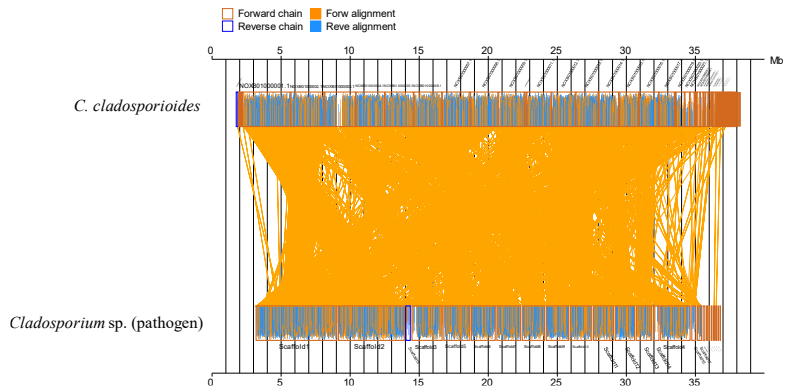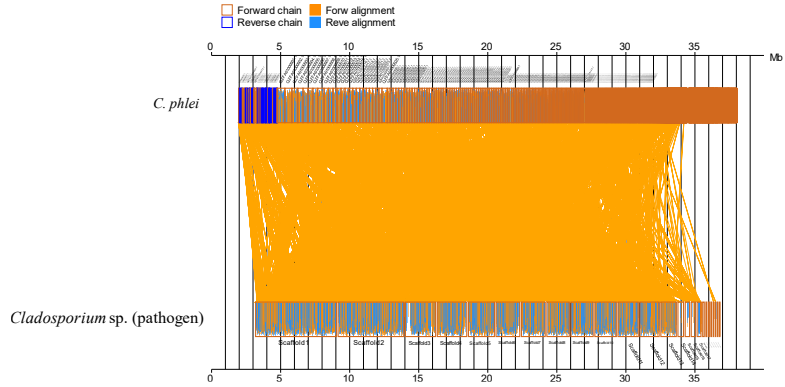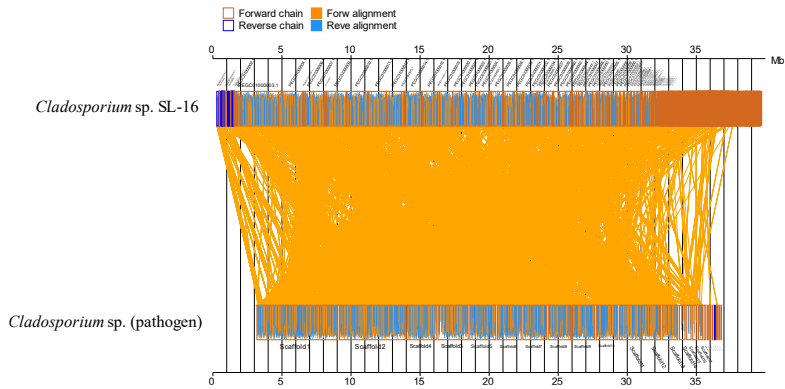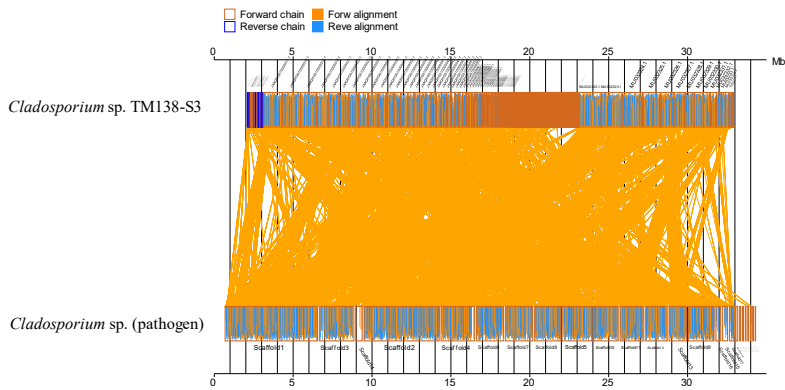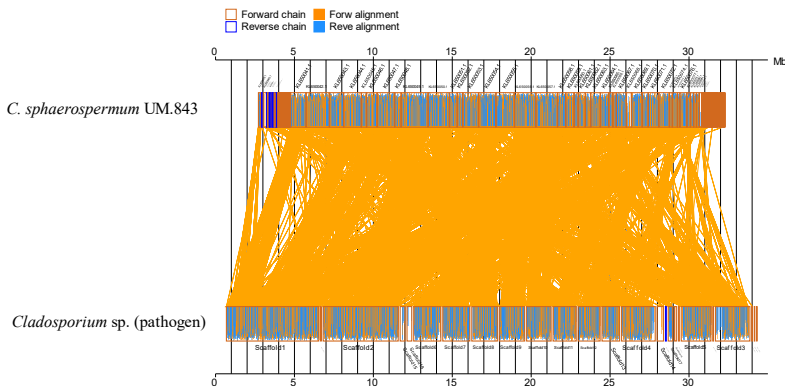

Supplement: Supplementary file 1 [file jof-08-00286-s001.zip › jof-1624251-supplementary/additional figures/Figure S8.pdf]

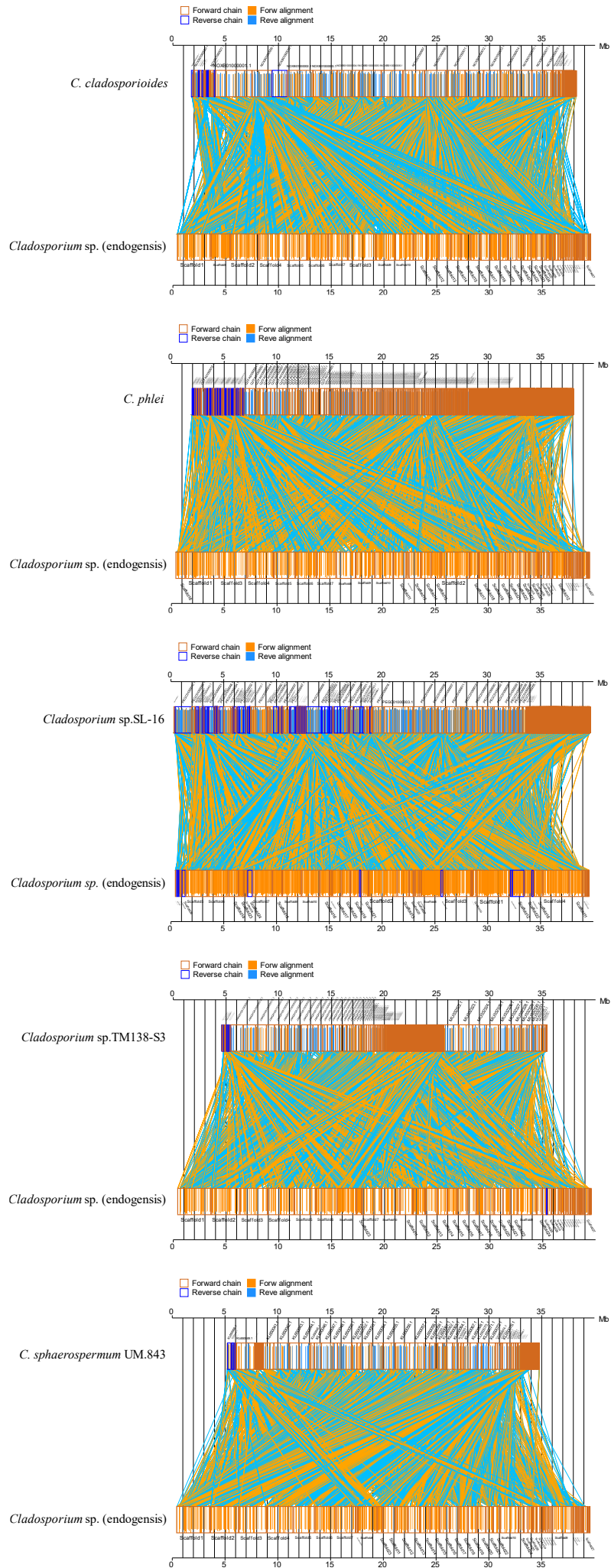

Supplement: Supplementary file 1 [file jof-08-00286-s001.zip › jof-1624251-supplementary/additional figures/Figure S9.pdf]
